# Simplified Chinese version of the Forgotten Joint Score (FJS) for patients who underwent joint arthroplasty: cross-cultural adaptation and validation

**DOI:** 10.1186/s13018-016-0508-5

**Published:** 2017-01-14

**Authors:** Shiqi Cao, Ning Liu, Wuxiang Han, Yunpeng Zi, Fan Peng, Lexiang Li, Qiwei Fu, Yi Chen, Weijie Zheng, Qirong Qian

**Affiliations:** Joint Surgery and Sports Medicine Department, Changzheng Hospital, Second Military Medical University, No. 415, Fengyang Road, Huangpu District, Shanghai, 200003 People’s Republic of China

**Keywords:** Forgotten Joint Score, Arthroplasty, Reliability, Validity, Quality of life

## Abstract

**Background:**

The Forgotten Joint Score (FJS) is a newly developed health-related quality of life (HRQoL) questionnaire designed to evaluate the awareness after total knee arthroplasty (TKA). This study cross-culturally adapted and psychometrically validated a simplified Chinese version of the FJS (SC-FJS).

**Methods:**

Cross-cultural adaptation was performed according to the internationally recognized guidelines. One-hundred and fifty participants who underwent primary TKA were recruited in this study. Cronbach’s α and intra-class correlations were used to determine reliability. Construct validity was analyzed by evaluating the correlations between SC-FJS and the Knee Injury and Osteoarthritis Outcome Score (KOOS) and the short form (36) health survey (SF-36).

**Results:**

Each of the 12 items was properly responded and correlated with the total items. SC-FJS had excellent reliability [Cronbach’s α = 0.907, intra-class correlation coefficient (ICC) = 0.970, 95% CI 0.959–0.978). Elimination of any one item in all did not result in a value of Cronbach’s α of <0.80. SC-FJS had a high correlation with symptoms (0.67, *p* < 0.001) and pain (0.60, *p* < 0.001) domains of KOOS and social functioning (0.66, *p* < 0.001) domain of SF-36, and it also moderately correlated with function in daily living (0.53, *p* < 0.001) and function in sport and recreation (0.40, *p* < 0.001) domains of KOOS, and physical subscale of SF-36 (0.49–0.53, *p* < 0.001) but had a low (*r* = 0.20) or not significant (*p* > 0.05) correlation with mental subscale of SF-36.

**Conclusions:**

SC-FJS demonstrated excellent acceptability, internal consistency, reliability, and construct validity, which can be recommended for patients who underwent joint arthroplasty in Mainland China.

**Electronic supplementary material:**

The online version of this article (doi:10.1186/s13018-016-0508-5) contains supplementary material, which is available to authorized users.

## Background

Total knee arthroplasty (TKA) has proven to be a successful and effective treatment for end-stage arthritis and other knee disorders [1, 2]. As TKA has been performed more than one million a year, the patients’ postoperative subjective perception should draw greater attention.

From the 1980s, a large body of researches have been devoted to the development of health-related quality of life (HRQoL) questionnaires [3]. HRQoL questionnaires are patient-based questionnaires filled by the patients themselves for a better understanding of their disorder severity and more appropriate therapeutic approach [4].

Although many scoring systems have been applied to patients in different countries and cultural background, this need has become more essential with the growing number of multicenter and multinational studies [3], which provide more statistic power of randomized controlled trials [5]. When one reliable, valid questionnaire is being used in populations with different cultures, it is necessary to test the psychometric properties of the questionnaire rather than simply translating the content to avoid bias involved in cultural variety [6, 7].

The Forgotten Joint Score (FJS) is a newly developed 12-item score introducing a new aspect of HRQoL, the patient’s ability to forget the artificial joint in everyday life [8]. The original English FJS is distinguished in brief expression, good reliability, and validity [8–10] and have been translated into three languages including French, Dutch, and Danish [9–11]. However, there is no FJS in the Chinese version for this population so far.

The purpose of this study was to translate and adapt the FJS into a simplified Chinese version (SC-FJS) and evaluate the psychometric properties of the SC-FJS in native Chinese-speaking patients who underwent TKA and the psychometric properties we tested including reliability and validity.

## Methods

### Patients and data collection

Between March 2015 and September 2015, 150 patients who underwent total knee arthroplasty at least 1 year before the evaluation were included in our study and completed two rounds of the questionnaires. Detailed demographic and clinical characteristics of the participants are listed in Table 1. The inclusion criteria were as follows: age >18 years with independent signing authority, literate native Chinese speakers, and patients who underwent TKA due to degenerative osteoarthritis. Participants were excluded for knee infection or traumatic osteoarthritis, revised total knee arthroplasty, history of spine surgery or any surgery in the recent 1 month, and other uncontrolled systematic disorders, such as diabetes mellitus, malignant tumor, or hepatitis. The population also needed to meet the standards proposed in the article by Terwee et al. [12] that stated that a study should include at least 100 patients for internal consistency analysis and 50 patients for floor or ceiling effects, reliability, and validity analysis. All included participants were required to sign informed consent, and the study was approved by the clinical research Ethics Committee of Changzheng Hospital, Shanghai, China.

**Table 1:** Demographic and clinical characteristics of the participants

Characteristics	Number or mean ± SD
Age (years)	68.1 ± 7.4
Range	47–86
Gender	Total (*N* = 150)
Female	118 (78.7%)
Male	32 (21.3%)
Side	
Right	69 (46.0%)
Left	81 (54.0%)
Time after primary surgery	28.0 ± 9.7
Range	12–94

Patients should provide demographic data regarding gender, year of age, side of surgery, and time after primary surgery at the first day approving to participate the study and then should finished SC-FJS, Knee Injury and Osteoarthritis Outcome Score (KOOS), and the short form (36) health survey (SF-36). Two weeks later, participants filled in SC-FJS for the second time to assess its test-retest reliability.

### Translation and cross-cultural adaptation

The steps of translation and transcultural adaptation followed previous guidelines in five steps, including forward translation, synthesis of the translation, backward translation, summarization of prefinal version, and determination of final version (Table 2) [3, 13]. Eventually, all researchers involved in this study discussed issues from the previous test and developed the final SC-FJS.

**Table 2:** Steps of translation and trans-cultural adaptation

Steps	Detailed contents
Forward translation	Two bilingual translators independently translated the metric from English to simplified Chinese. One of the translators was an orthopedic surgeon in the author’s hospital; the other one was a professional translator without medical background.
Synthesis of the translation	Two translators and other researchers unified contradictions regarding language expression and cultural difference after a consensus meeting and obtained the first SC-FJS.
Backward translation	Two native English speakers fluent in English, with medical background and blind to the previous original English version of FJS, independently translated the first SC-FJS back into the English version.
Summarization of prefinal version	A consensus meeting with all researchers including four forward and backward translators was held to resolve all discrepancies, ambiguities, or any other verbal issues to reach a prefinal SC-FJS.
Determination of final version	Researchers invited 20 patients underwent TKA to preliminarily test the prefinal version and collect feedbacks from them.

*FJS* Forgotten Joint Score, *SC-FJS* simplified Chinese version of the Forgotten Joint Score, *TKA* total knee arthroplasty

### Questionnaires

The FJS is a disease-specific questionnaire evaluating patients’ awareness of an artificial joint in everyday life [8]. In this 12-item questionnaire, all questions are answered in never, almost never, seldom, sometimes, mostly and “not relevant to me,” corresponding to 4 to 0 points and missing value, respectively. Total points are calculated according to the average score of all answered questions (eliminating missing value) and then multiplied by 25 into centesimal system (0–100 points). Higher scores refer to better outcome, that is, a better “forgotten” index of the joint and a low degree of awareness.

KOOS is a knee function score that mainly evaluates knee-related clinical symptoms and function. Consistent with FJS, the higher scores for KOOS refer to better function for patients [14]. SF-36 is a questionnaire assessing general quality of life [15]. Both of the scales above have been translated into Chinese and proved to possess good reliability and validity [16, 17].

### Psychometric assessments and statistical analysis

According to the original author’s proposal, if more than two items in SC-FJS were not answered, the questionnaire should be judged as invalid questionnaire and rejected in the final analysis [18].

To assess acceptability of SC-FJS, patients were asked for the difficulties encountered. Miss rates for each item were calculated, and if it was more than 5% for a certain item, it suggested that the acceptability and intelligibility were not satisfying [19]. Besides, mean completion time was obtained for all participants.

Statistical analysis for score distribution was performed. Floor and ceiling effects exceeding 15% were considered to be significant [12].

Reliability was examined in terms of test-retest reliability and internal consistency. The test-retest reliability was tested by comparing outcomes when the same patient without changes in health answered SC-FJS in two separated circumstances. It was evaluated by the intra-class correlation coefficient (ICC), which is derived from a two-way analysis of variance in a random effects model. ICC > 0.8 and >0.9 were considered as good and excellent reliability [20]. Meanwhile, Cronbach’s alpha was used to assess internal consistency of the questionnaire, and >0.7, 0.8, and 0.9 were considered as acceptable, good, and excellent internal consistency, respectively [12]. In addition, Bland-Altman plots were carried out to estimate systematic bias between the two measures [21].

Validity for SC-FJS was evaluated in dimensions of content validity and construct validity. To assess content validity of SC-FJS, we invited one rehabilitation expert and three orthopedic experts to analyze the correlation between content in each item and state of disease. Good construct validity meant that the questionnaire correlated well with measures of the same construct (convergent validity) while correlating poorly with measures of different constructs (divergent or discriminant validity) [22]. Based on this theory, we assumed that the score of SC-FJS should be in accordance with subdomains of KOOS and physical subdomains (physical function, role-physical, and bodily pain) of SF-36, but not with mental subdomains (role-emotional and mental health) of SF-36. Under this assumption, we calculated the Pearson’s correlation coefficient (*r*) between SC-FJS and domains of KOOS and SF-36. Then, the construct validity for SC-FJS was evaluated by comparing how data fitted with the calculated correlations, judged as poor (*r* = 0–0.2), fair (*r* = 0.2–0.4), moderate (*r* = 0.4–0.6), very good (*r* = 0.6–0.8), or excellent (*r* = 0.8–1.0) [22].

Statistical Package for the Social Sciences, version 20.0 (SPSS, Chicago, IL) was used to analyze the data. Mean values were reported with standard deviation (SD). ICC values were reported with 95% confidence intervals (CIs). *p* values of 0.05 or less were considered significant.

## Results

### Translation and cross-cultural adaptation process

There were no major problems in the forward and back translations of FJS. Some minor differences were found in some items due to the cultural diversity and then adapted cross-culturally, such as item 10, “Are you aware of your artificial knee when doing housework or gardening?”, which was a question about the daily housework. Seldom Chinese urban population owns a house with a garden, and most people living in the countryside are not used to gardening as a daily housework. Instead, the motor function related to daily household that patients who underwent TKA cared most was the ability to buy groceries around. Therefore, the item was adapted to be “Are you aware of your artificial knee when doing housework/buying groceries/farming?”. Furthermore, the background for item 10 was household. Some males might seldom do these in daily life owing to traditional culture, so we marked “assume” ahead of the original items to outline the hypothetical situation in case of omission.

In pilot trial, three out of ten patients mistakenly considered that items in prefinal SC-FJS were asking the frequency they were able to finish a corresponding activity with the knee which underwent TKA. Under the circumstances, we emphasized “aware of your artificial knee” at the beginning of the questionnaire in bold and underlined font and informed all participants of this content orally. Afterwards, no misunderstanding of the questionnaire turned up for the following participants (Additional file 1).

### Acceptability and score distribution

In formal study, no participants complained any content was too difficult to understand at the first time of completing SC-FJS. All items had an answer rate of 100%. The choice “not relevant to me,” regarded as missing value, however, was chosen for 2 (1.3%), 4 (2.7%), and 28 (18.7%) times in item 10, 11, and 12, respectively. The mean time to complete SC-FJS was 85 ± 23 s.

Absolute values of all three scores are listed in Table 3. No ceiling effect (2.0%) and floor effect (0%) were observed in the total score of SC-FJS.

**Table 3:** Absolute values of all scores

Scales	Mean ± SD	Minimum	Median	Maximum
FJS	60.7 ± 21.0	8	63	100
KOOS				
Symptoms	66.6 ± 17.9	11	66	100
Pain	72.7 ± 14.8	31	72	100
Function in daily living	68.9 ± 16.7	32	74	99
Function in sport and recreation	39.5 ± 26.9	0	40	100
Quality of life	52.9 ± 23.2	6	56	100
SF-36				
Physical functioning	52.1 ± 19.2	5	50	90
Pain	58.9 ± 21.8	10	62	100
Role physical	49.2 ± 29.5	0	50	100
General health	56.7 ± 23.9	0	60	95
Vitality	56.5 ± 20.9	10	55	90
Role emotional	53.8 ± 29.6	0	67	100
Mental health	60.7 ± 19.7	0	64	96
Social functioning	59.0 ± 23.3	0	56	100

*FJS* Forgotten Joint Score, *KOOS* Knee Injury and Osteoarthritis Outcome Score, *SF-36* short form 36

### Reliability

**Fig. 1:**
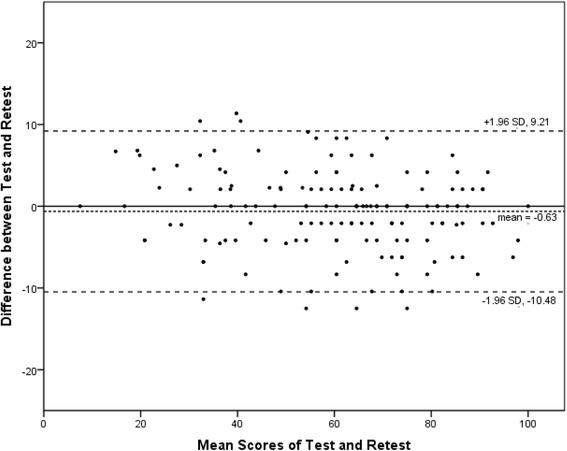
The Bland-Altman plot for test-retest agreement of SC-FJS. The differences between the scores for SC-FJS from the two test sessions were plotted against the mean of the test and retest. The *line* indicates mean difference value of the two sessions and the 95% (±1.96 standard deviation) limits of agreement

The internal consistency of SC-FJS was excellent (Cronbach’s alpha = 0.907). Elimination of one item in all 12 questions did not result in a value of <0.80. All items correlated with the total score of >0.47 (Table 4). The test-retest reliability of SC-FJS was also excellent. Mean score of the retest was 60.1 ± 20.0, which was comparable with the first test (60.7 ± 21.0). ICC for the overall SC-FJS was 0.970 (95%CI, 0.959–0.978), and test-retest reliability of each question was good or excellent (ICC = 0.86–0.95) (Table 4). Bland-Altman plots for the two measures revealed no systematic error (Fig. 1), which suggested good test-retest agreement and reproducibility of SC-FJS [21].

**Table 4:** Internal consistency and test-retest reliability of the SC-FJS

Item	Mean score ± SD	Item-total correlation	Alpha if item removed	ICC values (CIs range)
1	3.44 ± 0.81	0.601	0.902	0.91 (0.88–0.94)
2	3.37 ± 0.91	0.656	0.899	0.88 (0.83–0.91)
3	2.71 ± 1.20	0.670	0.897	0.88 (0.84–0.92)
4	2.73 ± 1.05	0.636	0.899	0.86 (0.81–0.90)
5	3.31 ± 0.93	0.683	0.898	0.89 (0.85–0.92)
6	2.33 ± 1.22	0.654	0.898	0.90 (0.86–0.93)
7	2.30 ± 1.15	0.640	0.899	0.92 (0.88–0.94)
8	1.89 ± 1.28	0.631	0.899	0.91 (0.87–0.93)
9	1.57 ± 1.40	0.650	0.899	0.93 (0.90–0.95)
10	2.02 ± 1.43	0.762	0.893	0.95 (0.93–0.97)
11	1.70 ± 1.45	0.678	0.897	0.91 (0.88–0.94)
12	1.76 ± 1.27	0.476	0.907	0.93 (0.90–0.95)

*SC-FJS* simplified Chinese version of Forgotten Joint Score, *ICC* intraclass correlation coefficient, *CIs* confidence intervals

### Validity

According to the evaluation of rehabilitation expert and orthopedic experts in SC-FJS, content validity was good for the questionnaire, and information derived from all questions was adequate to assess the function of patients who underwent TKA. Under this circumstance, it was not recommended to add to or remove any questions.

Table 5 lists the data of construct validity of SC-FJS, which were consistent with our consumption. The result revealed that SC-FJS correlated well with symptoms (0.67, *p* < 0.001) and pain (0.60, *p* < 0.001) domains of KOOS and social functioning (0.66, *p* < 0.001) domain of SF-36. The correlation between SC-FJS and function in daily living (0.53, *p* < 0.001) and function in sport and recreation (0.40, *p* < 0.001) domains of KOOS and physical subscale of SF-36 (0.49–0.53, *p* < 0.001) was also moderate. Meanwhile, SC-FJS was weakly (*r* = 0.20) or not significantly (*p* > 0.05) correlated to the mental subscale of SF-36. All of these suggested satisfied divergent or discriminant validity for SC-FJS.

**Table 5:** Construct validity of the simplified Chinese version of FJS

Scales	Correlation coefficient (*r*)^a^	*p* value
KOOS		
Symptoms	0.672^**^	<0.001
Pain	0.604^**^	<0.001
Function in daily living	0.532^**^	<0.001
Function in sport and recreation	0.402^**^	<0.001
Quality of life	0.297^**^	<0.001
SF-36		
Physical functioning	0.503^**^	<0.001
Pain	0.528^**^	<0.001
Role physical	0.494^**^	<0.001
General health	0.265^**^	<0.001
Vitality	0.292^**^	<0.001
Role emotional	0.204^*^	0.012
Mental health	0.086	0.296
Social functioning	0.662^**^	<0.001

The sample size for the analysis of construct validity was 150

*FJS* Forgotten Joint score, *KOOS* Knee Injury and Osteoarthritis Outcome Score, *SF-36* short form 36

^*^Correlation is significant at the 0.05 level (two-tailed)

^**^Correlation is significant at the 0.01 level (two-tailed)

^a^Calculated by the Pearson’s correlation of the simplified Chinese version of the FJS with KOOS and SF-36

## Discussion

HRQoL questionnaires are very important and valuable for clinical research, especially in the quantification of patients’ status of function and data analysis among studies. Nowadays, due to the largest population of patients and government’s greater attention to scientific research in China, a sharp increase in the quantity and quality of clinical research has been witnessed over years, and the second large number of papers is publishing annually [22]. Therefore, valid questionnaires are urgently needed to support this huge amount of clinical research.

There is no disease-specific questionnaire available in China that can be used to evaluate patients with the awareness of artificial joint after total joint arthroplasty, a common problem that imposes a considerable burden on the affected individuals and society. Only some functional questionnaires, such as KSS and WOMAC, for measurement of quality-of-life state of the upper extremity are available in a validated Chinese version [23, 24]. However, these questionnaires were not specifically developed for the subjective awareness of artificial joint and may be affected by the contralateral knee without surgery, other lower limb joints with poor function, and the spine. FJS, the questionnaire to evaluate the subjective perception for the joint after joint arthroplasty, has been translated into three languages and proven to be well reliable and valid [9–11]. In consequence, we considered that the cross-cultural adaptation for FJS in Chinese, the language spoken by the largest population around the world, was of great significance, which was the fundamental objective for the study.

In our study, the process of translation and cross-cultural adaptation went exceptionally well. No large modifications were made on the original questionnaire, and we just adjusted some items in terms of the cultural gap between Chinese and western cultures. After the adaptation, no question was hard to understand for participants, and all items were answered 100% in pretest and formal study, which revealed good acceptability of SC-FJS. Most participants in our study were elderly patients, and age and other organ functions highly restricted their athletic ability, which could be an explanation for the fact that 28 (18.7%) patients chose “not relevant to me” in item 12. The corrected item-total correlation (CITC) for item 12 was also much lower than other items (CITC = 0.476), which could be explained as follows. First, the rate of missing value for item 12, mostly chosen by patients with lower motor function, was high, and the score for this item was calculated as the average of the other scores, which very likely enhanced the score for item 12 compared to the true score of awareness when they had strenuous sports. Second, the purpose for some patients bearing TKA was to do their favorite sport, such as square dance, a famous sport with Chinese feature, and the delight mood could lower uncomfortable feelings when doing favorite sport. Last but not the least, favorite sport varied a lot in different individuals, and people with better motor function tended to like more vigorous sport, which made them easier to be aware of artificial joint. Under the circumstances, we suggested adding the choice “I can’t do this” to item 12 scoring 0.

SC-FJS showed perfect internal consistency (Cronbach’s alpha = 0.907) and test-retest reliability (ICC = 0.970), which was consistent with other cross-cultural adaptation studies and original study for FJS [9, 11, 25, 26]. The lowest ICC value presented to item 4 (“Are you aware of your artificial knee when taking a bath/shower?”, ICC = 0.86). One possible reason might be the subjective feeling of pain changing in different situations, even if the disease itself remained firm in 2 weeks. The highest ICC value, however, was presented in item 10 (“Are you aware of your artificial knee when doing housework/buying groceries/farming?”, ICC = 0.95), a question based on objective fact, which indirectly revealed the success of cross-cultural adaptation. Furthermore, the 2-week gap for the two separated evaluation of questionnaire was appropriate, according to studies published previously, that the knee function reaches plateau beyond 1 year after TKA [27, 28], which avoided possible error.

The correlation between SC-FJS and domains of KOOS, as well as SF-36, was in accordance with our hypothesis. The association between SC-FJS and symptoms and pain domains of KOOS was the strongest in our study (*r* = 0.672 and 0.604, respectively; good). One possible reason might be that these two domains of KOOS and FJS were both designed for evaluation of joint function and symptoms, and symptoms and pain are both important causes for patients getting aware of the artificial joint. Likewise, function in daily living and sport and recreation domains of KOOS and physical subscales of SF-36 were moderately correlated with SC-FJS (*r* = 0.40–0.53). The correlation, however, was weaker than symptoms and pain domains of KOOS, perhaps because the two domains of KOOS indicated specific situation instead of specific sensation, which were not as direct as symptoms and pain; and SF-36 was a scale for general situation and showed less accuracy than other specific scales [29]. In addition, mental subscales of SF-36 were weakly associated with SC-FJS (*r* = 0.20 or *p* > 0.05), which could be well understood as psychological state was affected by many factors other than physical situation.

There are several limitations in our study. First, the sample was limited in size and may not fully represent the Chinese population. Second, although simplified Chinese is the official language in China, China is a country with multiple nationalities, most of which have their own language. Thus, the problem of national cultural differences should be noted. Finally, the authors did not evaluate the responsiveness of SC-FJS, which could be carried out in the follow-up studies.

## Conclusions

In summary, FJS was successfully translated and cross-culturally adapted into simplified Chinese. The SC-FJS demonstrated good reliability and validity in the evaluation of mainland Chinese patients with history of TKA. Suggestion for improvement of FJS would be adding the choice “I can’t do this” to item 12 scoring 0. The FJS seems promising, and this work will greatly promote the use of FJS by physicians and researchers in Mainland China in data collection.

## Additional file

Additional file 1: Simplified Chinese version of the Forgotten Joint Score. (DOCX 18 kb)

## Abbreviations

CIs: Confidence intervals
CITC: Corrected item-total correlation
FJS: Forgotten Joint Score
HRQoL: Health-related quality of life
ICC: Intra-class correlation coefficient
KOOS: Knee injury and osteoarthritis outcome score
SC-FJS: Simplified Chinese version of the Forgotten Joint Score
SD: Standard deviation
SF-36: The short form (36) health survey
TKA: Total knee arthroplasty
