# Supplementary material for: Simplified Chinese version of the Forgotten Joint Score (FJS) for patients who underwent joint arthroplasty: cross-cultural adaptation and validation
Source: J Orthop Surg Res. 2017 Jan 14;12:6. doi: 10.1186/s13018-016-0508-5 (PMC5237477; doi:10.1186/s13018-016-0508-5)
Supplement: Additional file 1: — Simplified Chinese version of the Forgotten Joint Score. (DOCX 18 kb) [file 13018_2016_508_MOESM1_ESM.docx]

**FJS-12评分**

假设您在做以下事情或类似的活动，您会**感受到您的关节是人工材料**吗？（该量表并非询问您从事以下动作的频率）

| 1. 睡觉 | ①从来没有 ②几乎没有 ③很少 ④有时 ⑤经常 ⑥与我无关 |
| --- | --- |
| 1. 坐超过1小时 | ①从来没有 ②几乎没有 ③很少 ④有时 ⑤经常 ⑥与我无关 |
| 1. 步行超过15分钟 | ①从来没有 ②几乎没有 ③很少 ④有时 ⑤经常 ⑥与我无关 |
| 1. 洗澡 | ①从来没有 ②几乎没有 ③很少 ④有时 ⑤经常 ⑥与我无关 |
| 1. 坐车 | ①从来没有 ②几乎没有 ③很少 ④有时 ⑤经常 ⑥与我无关 |
| 1. 上下楼梯 | ①从来没有 ②几乎没有 ③很少 ④有时 ⑤经常 ⑥与我无关 |
| 1. 走在高低不平的路上 | ①从来没有 ②几乎没有 ③很少 ④有时 ⑤经常 ⑥与我无关 |
| 1. 从坐着站起来 | ①从来没有 ②几乎没有 ③很少 ④有时 ⑤经常 ⑥与我无关 |
| 1. 长时间站立 | ①从来没有 ②几乎没有 ③很少 ④有时 ⑤经常 ⑥与我无关 |
| 10、 做家务或买菜 | ①从来没有 ②几乎没有 ③很少 ④有时 ⑤经常 ⑥与我无关 |
| 11、 散步或徒步旅行 | ①从来没有 ②几乎没有 ③很少 ④有时 ⑤经常 ⑥与我无关 |
| 12、 进行最喜爱的体育活动 | ①从来没有 ②几乎没有 ③很少 ④有时 ⑤经常 ⑥与我无关 |
